# Genome Sequences of Two Emerging Non-O157 Shiga Toxin-Producing *Escherichia coli* Strains

**DOI:** 10.1128/genomeA.00200-13

**Published:** 2013-05-16

**Authors:** Guojie Cao, Wenting Ju, Lydia Rump, Shaohua Zhao, Likou Zou, Charles Wang, Errol Strain, Yan Luo, Ruth Timme, Marc Allard, Eric Brown, Jianghong Meng

**Affiliations:** Department of Nutrition and Food Science, University of Maryland, College Park, Maryland, USAa;; Joint Institute for Food Safety and Applied Nutrition (JIFSAN), University of Maryland, College Park, Maryland, USAb;; Division of Animal and Food Microbiology, Office of Research, Center for Veterinary Medicine, Food and Drug Administration, Laurel, Maryland, USAc;; Laboratory of Microbiology, Sichuan Agricultural University, Dujiangyan, Sichuan, Chinad;; Division of Microbiology, Center for Food Safety and Applied Nutrition, Food and Drug Administration, College Park, Maryland, USAe;; Biostatistics Branch, Center for Food Safety and Applied Nutrition, Food and Drug Administration, College Park, Maryland, USAf

## Abstract

Shiga toxin-producing *Escherichia coli* (STEC) causes severe illness in humans, including hemorrhagic colitis and hemolytic uremic syndrome. A parallel evolutionary model was proposed in which *E. coli* strains of distinct phylogenies independently integrate Shiga toxin-encoding genes and evolve into STEC. We report the draft genomes of two emerging non-O157 STEC strains.

## GENOME ANNOUNCEMENT

Shiga toxin-producing *Escherichia coli* (STEC) has become a significant food-borne pathogen since *E. coli* O157:H7 was first identified as the agent causing food-borne outbreaks in 1982 (1). Non-O157 STEC has been increasingly associated with food-borne illness (2–5) and accounts for approximately 50% of all STEC infections in the United States (4). We selected two STEC strains of serogroups O26 and O111 for whole-genome sequencing (WGS) analysis (6). It was proposed that STEC strains from multiple lineages have acquired Shiga toxin genes (*stx*) independently, referred to as a parallel evolutionary process (7, 8).

There are approximately 100 genome sequences of STEC that are deposited in GenBank. We report two draft genome sequences of non-O157 STEC strains: *E. coli* O26:H11 strain CFSAN001629 (host, human; *stx* gene type, *stx*_1_) and *E. coli* O111:H8 strain CFSAN001632 (host, human; *stx* gene type, *stx*_1_). The genome sequence data bring more clarity to the evolutionary processes and virulence factors of these important pathogens.

The two STEC strains were sequenced using a 454 FLX+ pyrosequencing system (Roche, Branford, CT) to obtain 20 to 24×-coverage draft genome sequences. Genomic DNA from each strain was extracted from overnight Trypticase soy broth (TSB) culture with a DNeasy blood and tissue kit (Qiagen, Valencia, CA). Genomic contigs were assembled (*de novo*) with the 454 Life Sciences Newbler software package version 2.6 (Roche). The data for each draft genome sequence are as follows: for CFSAN001629, 24× coverage, 275 contigs, genome size of 5,444,981 bp, and contig *N*_50_ of 100,856 bp, and for CFSAN001632, 20× coverage, 259 contigs, genome size of 5,303,432 bp, and contig *N*_50_ of 99,424 bp. Sequences were annotated with the NCBI Prokaryotic Genomes Automatic Annotation Pipeline (9). Totals of 5,564 and 5,400 genes were identified for strains CFSAN001629 and CFSAN001632, respectively.

A detailed report of the phylogenetic analyses of these two draft sequences will be included in a future publication.

### Nucleotide sequence accession numbers.

The draft genome sequences of *E. coli* O26:H11 CFSAN001629 and *E. coli* O111:H8 CFSAN001632 are available in GenBank under accession no. AMXO00000000 and AMXQ00000000, respectively.
